# Efficacy and Safety of the Arsenic Trioxide/Lipiodol Emulsion in the Transcatheter Arterial Chemoembolization Combined with Apatinib in the Treatment of Advanced Hepatocellular Carcinoma

**DOI:** 10.1155/2021/5565793

**Published:** 2021-08-19

**Authors:** Zhaonan Li, Quanjing Chen, Wenguang Zhang, Guangyan Si, Jing Li, Dechao Jiao, Xinwei Han

**Affiliations:** ^1^Department of Interventional Radiology, First Affiliated Hospital of Zhengzhou University, Zhengzhou 450000, China; ^2^Department of Laboratory Medicine, Hospital of Chengdu University of Traditional Chinese Medicine, Chengdu 610000, China; ^3^Department of Interventional Radiology, The Affiliated Hospital of Traditional Chinese Medicine of Southwest Medical University, Luzhou 646000, China

## Abstract

**Purpose:**

The goal of this study was to assess the clinical efficacy and safety of the arsenic trioxide (ATO)/lipiodol emulsion in the transcatheter arterial chemoembolization (TACE) combined with apatinib in the treatment of advanced hepatocellular carcinoma (HCC).

**Methods:**

From December 2015 to February 2017, a total of 87 patients were consecutively enrolled and underwent ATO-TACE (aTACE) combined with apatinib in the treatment of advanced HCC. The treatment response and adverse events were assessed at the first month and third month after aTACE therapy. Progression-free survival (PFS), overall survival (OS), and treatment-related adverse events were also analyzed.

**Results:**

87 patients (57 men; 30 women) were enrolled in the present study. Compared to that at the pre-aTACE examination, the levels of AST and ALT were elevated at the first week after procedure (65.84 U/L ± 22.93 U/L vs. 54.15 U/L ± 19.60 U/L, *p*=0.032; 63.44 U/L ± 22.50 U/L vs. 51.60 U/L ± 13.89 U/L, *p*=0.027, respectively). Most of the adverse events were grade 1 or 2 according to National Cancer Institute Common Terminology Criteria for Adverse Event (CTCAE). Of the exception, 4 persons (2%) did have grade 3 hand-foot skin reactions, 1 (1%) had grade 3 diarrhea, 1 (1%) had grade 3 hypertension, and 3 (3%) had grade 3 proteinuria and forced to reduce the dose of apatinib by half. The survival analysis of the combination with aTACE and apatinib therapy found that the median PFS was 10.2 months (95% CI: 8.543–11.857), and the median OS was 23.300 months (95% CI: 20.833–25.767). Additionally, both univariate and multivariate Cox regression revealed that the tumor burden (≤50%) and the patients without portal vein tumor thrombus (PVTT) significantly impacted the patient's PFS and OS and were related to better survival.

**Conclusion:**

aTACE combined with apatinib is a safe and promising treatment approach for patients with advanced HCC. Additionally, tumor burden (≤50%) and the patients without PVTT are associated with better PFS and OS.

## 1. Introduction

Hepatocellular carcinoma (HCC) is the fourth leading cause of cancer deaths worldwide, with approximately 84,000 newly diagnosed cases and 78,000 deaths each year [1]. Most of the clinically diagnosed HCC have lost the opportunity for surgery [2, 3]. Transcatheter arterial chemoembolization (TACE) is a major palliative treatment used for unresectable HCC, and many clinical institutions support the use of TACE in early and advanced HCC patients [4]. However, not entirely destroying the tumors after TACE, which resulted from the release of angiogenic cytokines from tumor cells after chemoembolization, are the leading shortcomings of TACE in the management of HCC. Besides, repeated TACE treatment would aggravate liver function and induce local ischemia and hypoxia environment, resulting in increased expression of the hypoxia-inducible factor and vascular endothelial growth factor in HCC and eventually leading to tumor recurrence. In fact, tumor angiogenesis facilitates the supply of oxygen and nutrients to cells and therefore plays an essential role in tumor recurrence, development, and metastasis.

Arsenic trioxide (ATO) is the main component of arsenic and has been mostly used intravenously to treat solid tumors [5]. Of note, ATO can effectively reduce the invasion, angiogenesis, and self-renewal of HCC cells to play its anticancer effect on HCC [6, 7]. Furthermore, ATO can also target 14-3-3 *η*/NF-kB feedback loop that reverses chemoresistance of HCC. These studies have revealed the critical role of ATO in the treatment of HCC, and the application of ATO can help to improve the therapeutic effect of this disease [8, 9]. Additionally, apatinib is a potent vascular endothelial growth factor receptor 2 (VEGFR-2) inhibitor, which can inhibit tumor neovascularization after TACE and play an anticancer role by effectively binding and inhibiting VEGFR-2 [10, 11]. The combination of two antiangiogenic treatment strategies may reduce the local tumor recurrence time and improve patient survival. Studies have reported that the median overall survival (OS) of the apatinib combined with the TACE group was significantly improved compared with the TACE alone. Similarly, apatinib plus TACE significantly prolonged median progression-free survival (PFS) compared with TACE monotherapy [12–14]. To date, no study has focused on ATO-TACE (aTACE) combined with apatinib. Therefore, the purpose of this study was to evaluate the safety and efficacy of aTACE combined with apatinib administration for the treatment of advanced HCC.

## 2. Materials and Methods

### 2.1. Patients

This was a retrospective cohort study conducted in a single-center approved by the institutional review board. In this retrospective study, we included 87 patients (53.1 ± 12.4 years; range 35–71 years) who received aTACE combined with apatinib in the treatment of advanced HCC. The inclusion and exclusion criteria are listed in Table 1.

### 2.2. Procedure

#### 2.2.1. aTACE Treatment

All aTACE procedures were performed by two interventional radiologists (with more than ten years of experience in interventional radiology) to ensure consistency. Hepatic artery angiography was performed using a 5 Fr catheter to identify the tumor and feeder(s). Then, superselective catheterization of the feeding artery was performed using a 2.0 F microcatheter (Progreat, Terumo Corporation, Tokyo, Japan). Arsenic trioxide (20 mg) was diluted in 0.9% NaCl, mixed with a maximum of 20 mL iodized oil (lipiodol) per session. Finally, after lipiodol was evenly deposited in the tumor, the artery feeding the tumor was utterly embolized with microspheres (100–300 *μ*m; Jiangsu Hengrui Medicine Co. Ltd., Jiangsu, China). The embolization extent was determined according to the tumor size and the patients' liver function.

### 2.3. Apatinib Administration

Each patient in this study was initially suggested to take apatinib at 500 mg, and apatinib was orally taken 3–5 days after TACE treatment. According to the National Cancer Institute Common Terminology Criteria for Adverse Events (version 4.0), if some patients did not tolerate the drug well or if adverse reactions occurred, the dose of apatinib was reduced to 250 mg to relieve the adverse events. If the adverse reactions (≥grade 3) continued to occur after dose adjustment or the adverse events of gastrointestinal bleeding related to apatinib occur, the drug administration would be suspended temporarily. When the adverse event was relieved or eliminated, the dose would return to 250 mg. Each cycle of apatinib oral administration lasted 28 days.

### 2.4. Follow-Up

All of the patients underwent laboratory tests including blood testing, contrast-enhanced computed tomography (CT) prior to treatment, and at follow-up. Treatment response was evaluated using the modified Response Evaluation Criteria in Solid Tumors (mRECIST 2020 edition). Parameters such as serum albumin (ALB), total bilirubin (TBIL), alanine aminotransferase (ALT), and aspartate aminotransferase (AST) were tested to evaluate the liver function before the aTACE procedure (M0), at the first week after the first cycle of TACE (W1), at the first month after the first cycle of aTACE (M1), and at the third month after the first cycle (M3). Adverse events were assessed in accordance with the National Cancer Institute Common Toxicity Criteria for Adverse Events version 3.0.

### 2.5. Statistical Analysis

Statistical analysis was conducted using the statistical software SPSS 22.0 (SPSS Inc., Chicago, IL, USA). Categorical variables are expressed as numbers or percentages (%), and continuous variables are expressed as the mean ± standard deviation (SD). Kaplan–Meier survival curves were used for survival analysis. Univariate and multivariate Cox proportional hazards regression analyses were used to predict prognostic factors of progression-free survival (PFS) and OS. A *p* < 0.05 was considered for significant differences.

## 3. Results

### 3.1. Patient Characteristics

A total of 87 patients (57 men, 30 women) were enrolled in the present study. The mean age of the patients was 53.1 ± 12.4 years (range, 35–71 years). Of the 87 patients, 34 (39%) patients were younger than 60 years old, 57 (66%) patients were male, and 56 (64%) patients also had hepatitis B. The number of patients with Child–Pugh class A and B HCC was 52 (60%) and 35 (40%), respectively. There were 34 patients (39%) with an Eastern Cooperative Oncology Group performance status of 1. There were 27 patients (31%) with portal vein tumor thrombosis. In the whole therapy process, 78 (90%) patients received a dose of 500 mg apatinib for oral administration. Only nine patients (10%) had a reduction to 250 mg due to the drug not being well-tolerated. The tumor burden of 52 patients (60%) was equal to or less than 50%. The baseline characteristics of the patients are listed in Table 2.

### 3.2. Safety

**Figure 1:**
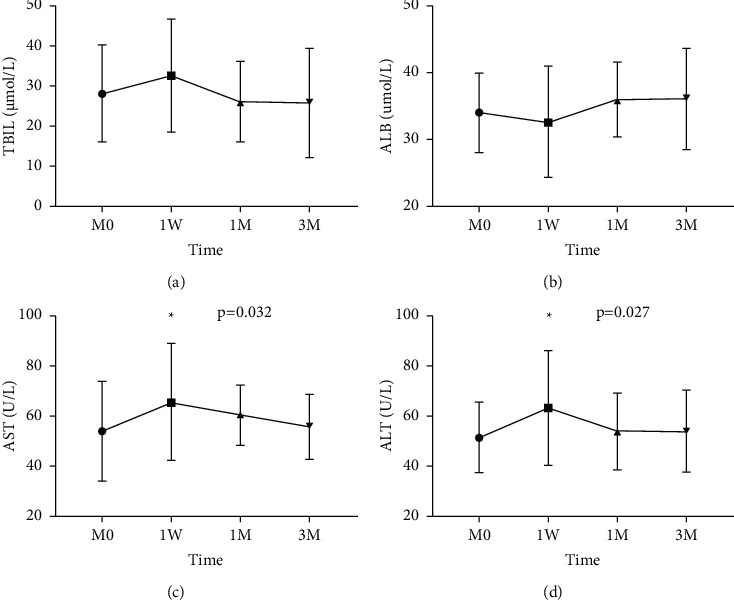
Changes in liver function at different times. (a) TBIL increased rapidly at 1 W after treatment and returned to the original level within 3 M. (b) In comparison with M0, the level of ALB was reduced slightly at 1 W, but there is an increase in ALB levels after 3 months of treatment. (c), (d) At 1 W, the levels of AST and ALT increased significantly but decreased to the original level at 1 M and 3 M. M0, pretreatment; 1 W, the first week after treatment; 1 M, the first month after treatment; 3 M, the third month after treatment.

All patients completed liver laboratory tests within 1 week, 1 month, and 3 months after the procedure. Compared to that at the pre-TACE examination, the levels of AST and ALT were elevated at the first week after the procedure (65.84 U/L ± 22.93 U/L vs. 54.15 U/L ± 19.60 U/L, *p*=0.032; 63.44 U/L ± 22.50 U/L vs. 51.60 U/L ± 13.89 U/L, *p*=0.027, respectively), and the TBIL level increased slightly one week (1 W) after the procedure and was controlled to normal levels after three months (3 M). Additionally, the level of ALB was reduced slightly at first one month (1 M), but there is an increase in ALB levels after three months of treatment (Figure 1).

### 3.3. Treatment-Related Adverse Events

Most of the adverse events were grade 1 or 2 according to National Cancer Institute Common Terminology Criteria for Adverse Event (CTCAE) (mild symptoms, no or local/noninvasive intervention indicated) (Table 3). The treatment-related adverse events included postembolization syndrome and apatinib-related adverse reactions. Common adverse reactions, such as abdominal pain, fever, vomiting, and increased ALT/AST, and apatinib-related reactions, such as hand-foot skin reactions, fatigue, hypertension, diarrhea, proteinuria, and oral ulcers, were predominantly mild. Besides, two patients developed grade 3 abdominal pain, which was relieved after timely clinical treatment. No toxin-induced death occurred in this study. Of the 87 patients, 4 people (2%) had grade 3 hand-foot skin reactions, 1 (1%) had grade 3 diarrhea, 1 (1%) had grade 3 hypertension, and 3 (3%) had grade 3 proteinuria who were forced to reduce the dose of apatinib by half.

### 3.4. PFS and OS

**Figure 2:**
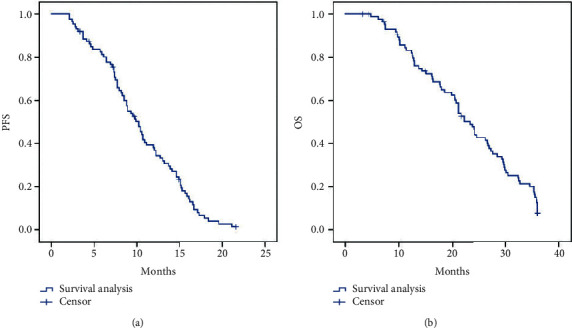
Median PFS and OS in patients who underwent combination of aTACE and apatinib therapy. (a) Median PFS was 10.2 months (95% CI: 8.543–11.857 months) in all subjects. (b) Median OS was 23.300 months (95% CI: 20.833–25.767 months). PFS, progression-free survival; OS, overall survival.

**Figure 3:**
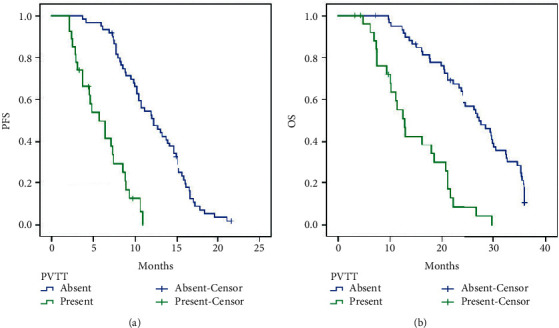
(a) Comparison of PFS between patients with PVTT (present) and without PVTT (absent) after the combination of aTACE and apatinib therapy. The median PFS was 5.700 months (95% CI: 3.543–11.857) for treatment with PVTT versus 12.200 months (95% CI: 9.733–14.667) for treatment without PVTT (*p* < 0.001, log-rank test). (b) Comparison of OS between patients with PVTT (present) and without PVTT (absent) after the combination of aTACE and apatinib therapy. The median OS was 12.800 months (95% CI: 10.447–15.153) for treatment with PVTT versus 27.300 months (95% CI: 22.393–32.207) for treatment without PVTT (*p* < 0.001, log-rank test). The 1-, 2-, and 3-year OS rates in patients with PVTT were 55.1%, 8.5%, and 0.0%, respectively, and the 1-, 2- and 3-year OS rates in patients without PVTT were 94.9%, 63.8%, and 10.6%, respectively. PFS, progression-free survival; OS, overall survival; aTACE, arsenic trioxide transcatheter arterial chemoembolization; PVTT, portal vein tumor thrombus.

**Figure 4:**
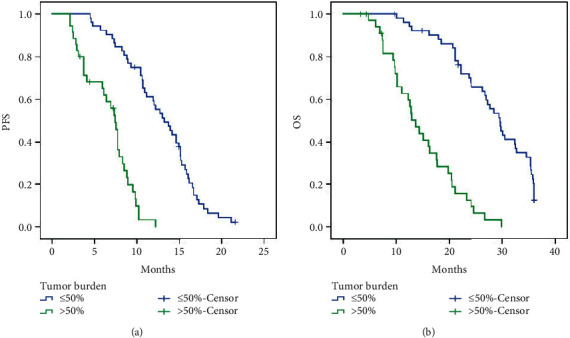
(a) Comparison of PFS between patients with tumor burden ≤50% and tumor burden >50% after the combination of aTACE and apatinib therapy. The median PFS was 7.400 months (95% CI: 6.704–8.096) for patients with tumor burden >50% versus 13.200 months (95% CI: 11.000–15.400) for tumor burden ≤50% (*p* < 0.001, log-rank test). (b) Comparison of OS between patients with tumor burden ≤50% and tumor burden >50% after the combination of aTACE and apatinib therapy. The median OS was 13.700 months (95% CI: 11.355–16.045) for patients with tumor burden >50% versus 29.600 months (95% CI: 26.881–32.319) with tumor burden ≤50% (*p* < 0.001, log-rank test). The 1-, 2-, and 3-year OS rates in patients with tumor burden >50% were 62.7%, 12.5%, and 0.0%, respectively, and the 1-, 2-, and 3-year OS rates in patients with tumor burden ≤50% were 96.1%, 70.0%, and 12.3%, respectively. PFS, progression-free survival; OS, overall survival; aTACE, arsenic trioxide transcatheter arterial chemoembolization.

The survival analysis of the combination with aTACE and apatinib therapy found that the median PFS was 10.2 months (95% CI: 8.543–11.857) (Figure 2(a)) and the median OS was 23.300 months (95% CI: 20.833–25.767) (Figure 2(b)). Comparison of PFS is performed between patients with portal vein tumor thrombus (PVTT) and without PVTT after combining aTACE and apatinib therapy. The median PFS was 5.700 months (95% CI: 3.543–11.857) for treatment with PVTT versus 12.200 months (95% CI: 9.733–14.667) without PVTT (*p* < 0.001, log-rank test) (Figure 3(a)). The median OS was 12.800 months (95% CI: 10.447–15.153) for treatment with PVTT versus 27.300 months (95% CI: 22.393–32.207) for treatment without PVTT (*p* < 0.001, log-rank test). The 1-, 2-, and 3-year OS rates in patients with PVTT were 55.1%, 8.5%, and 0.0%, respectively, and the 1-, 2-, and 3-year OS rates in patients without PVTT were 94.9%, 63.8%, and 10.6%, respectively (Figure 3(b)). Furthermore, comparison of PFS is performed between patients with tumor burden ≤50% and tumor burden >50% after the combination of aTACE and apatinib therapy. The median PFS was 7.400 months (95% CI: 6.704–8.096) for patients with tumor burden >50% versus 13.200 months (95% CI: 11.000–15.400) for tumor burden ≤50% (*p* < 0.001, log-rank test) (Figure 4(a)). The median OS was 13.700 months (95% CI: 11.355–16.045) for patients with tumor burden >50% versus 29.600 months (95% CI: 26.881–32.319) with tumor burden ≤50% (*p* < 0.001, log-rank test). The 1-, 2-, and 3-year OS rates in patients with tumor burden >50% were 62.7%, 12.5%, and 0.0%, respectively, and the 1-, 2-, and 3-year OS rates in patients with tumor burden ≤50% were 96.1%, 70.0%, and 12.3%, respectively (Figure 4(b)).

### 3.5. Factors Affecting PFS and OS

Univariate Cox proportional hazard regression indicated that age (>60 vs. ≤60), the Child–Pugh (A vs. B), liver cirrhosis (yes vs. no), AFP level (≤400 ng/mL vs. >400 ng/mL) and the HBV infection (yes vs. no) were not associated with longer PFS and OS (both *p* > 0.05). Additionally, both univariate and multivariate Cox regression revealed that the tumor burden (≤50%) and the patients without PVTT did have a significant impact on the patient's PFS and OS and was related to better survival (Table 4).

## 4. Discussion

As basic and clinical research has deepened, the use of ATO in the treatment of malignant tumors has recently attracted attention, such as HCC, breast cancer, and renal cell carcinoma [6, 15, 16]. ATO has been considered as a palliative treatment method for late-staged HCC patients in China [17–19]. The anticancer effect of ATO is mainly through upregulating the apoptosis promoter gene BAX and downregulating the apoptosis inhibitor gene BCL-2 to induce tumor cell apoptosis. More importantly, ATO could interrupt the telomerase activity of HCC cells and inhibit the vascular endothelial growth factor (VEGF), reducing tumor angiogenesis [20, 21]. Due to the favorable antitumor efficiency of ATO, it has been confirmed for treating late-staged HCC. However, the systemic ATO treatment brings in serious adverse events (including gastrointestinal bleeding, ventricular arrhythmia, and renal failure).The application of ATO in the management of HCC is still restrained. Recently, ATO/lipiodol emulsion in the transcatheter arterial chemoembolization might be a favorable treatment strategy to improve the efficacy of ATO while decreasing its systemic adverse events in HCC [22]. Duan et al. [23] used ATO-loaded CalliSpheres beads to treat unresectable HCC and found that compared with conventional TACE (cTACE) in treating unresectable HCC, ATO combined with TACE is more effective and has equally tolerance.

In order to improve the response of the tumor, this study is based on the use of ATO and sequential apatinib combination therapy, hoping to utilize two antiangiogenic agents to further control tumor recurrence and metastasis, so as to improve patient's survival. In fact, apatinib is a powerful inhibitor of VEGFR-2. As a highly selective VEGFR-2 blocker, the affinity of apatinib to VEGFR-2 tyrosine kinase is ten times greater than that of sorafenib [24, 25]. A meta-analysis [26] indicated that compared with TACE alone, TACE plus apatinib pronouncedly improved the half-year and one-year survival rate, disease control rate, and objective response rate of patients with advanced HCC. A single-center randomized controlled trial found that in comparison with patients who received TACE alone, patients treated with TACE plus apatinib had a higher objective response rate (ORR) at 9 and 12 months after treatment [27]. Furthermore, among HCC patients with the BCLC-C stage, Chen et al. demonstrated [28] that the ORR of the TACE combined with apatinib therapy was remarkably higher than that of the TACE alone at 1 and 3 months after treatment (66.7% vs. 39.6% and 45.8% vs. 17.6%, respectively). Our previous studies have illustrated that TACE combined with apatinib is a safe and promising treatment approach for patients with large HCC [29].

The efficacy of TACE combined with apatinib has been supported by several studies, whether the combination therapy of aTACE + apatinib is sufficient to achieve better therapeutic effects. Actually, compared with TACE plus apatinib, although the complications in this study are similar to those reported by Liu et al. [30], aTACE combined with apatinib has more advantages in PFS (10.2 months vs. 9.5 months) and OS (23.3 months vs. 22.0 months) [31]. Moreover, the univariate Cox regression analysis indicated no considerable correlation between median PFS or median OS with age, Child–Pugh, cirrhosis, HBV infection, and AFP level. To better analyze factors that independently predict survival in patients with advanced HCC, the combined results of univariate regression analysis and multivariate regression analysis pointed out that tumor burden and PVTT are the main prognostic factors affecting patients' PFS and OS. The study further revealed that tumor burden ≤50% and patients without PVTT were closely related to better PFS and OS. This conclusion is similar to the findings of Fan et al. [31], that is, PVTT involving the main portal vein is an independent predictor of OS. Furthermore, adverse events include postembolization syndrome and apatinib-related adverse reactions. The targeted molecular agents were controllable and safe in the management of HCC, and most of the toxicities observed in this study were classified as grade 1-2 adverse events. Only 10.3% (9/87) of patients had apatinib-related grade 3 toxicity. Of note, all serious adverse events were effectively relieved after symptomatic treatment.

Admittedly, the research has several significant limitations. First, the results of this study are limited by the small sample size and short follow-up time. Moreover, combining these two antiangiogenic therapeutic strategies could lead to an enhanced cellular response to hypoxia, thereby aggravating the disease and developing a more aggressive tumor phenotype. However, the relevant research is still in the blank stage, and the next step is to verify the two antiangiogenesis treatment strategies in cell and animal experiments. In addition, single-center retrospective studies may be affected by subjective selection bias. Therefore, a multicenter, randomized, controlled study with a larger sample size is needed to evaluate further the efficacy and safety of the combination of aTACE and apatinib in the treatment of advanced HCC.

## 5. Conclusion

In conclusion, aTACE combined with apatinib displayed favorable safety and effectiveness in the treatment of advanced HCC. The adverse events of apatinib need to be monitored during the application, despite the manageable appearance. Additionally, tumor burden (≤50%) and the patients without PVTT are associated with better PFS and OS.

## Figures and Tables

**Table 1:** Inclusion and exclusion criteria.

Inclusion criteria	Exclusion criteria
(1) Age range: 18–75 years	(1) Age <18 or >75 years
(2) HCC diagnosed according to EASL standards	(2) No pathology or image evidence
(3) Child–Pugh grade A or B	(3) Child–Pugh grade C
(4) HCC in BCLC stage C	(4) Patients with complete occlusion of the main portal vein
(5) ECOG score ≤2	(5) Patients with moderate or severe ascites
(6) The expected survival time >3 months	(6) Patients with serious comorbidities
	(7) The expected survival time ≤3 months
	(8) Patients who received other therapies during this study

EASL, European Association for the Study of the Liver; ECOG, Eastern Cooperative Oncology Group; HCC, hepatocellular carcinoma.

**Table 2:** Patient characteristics.

Characteristics	Patients (*n* = 87)		Percentage (%)
Age (mean, range)	53.1 ± 12.4 (35–71)		
≥60	53		61
<60	34		39

Sex			
Male	57		66
Female	30		34

ECOG performance status			
0	53		61
1	34		39

HBV infection			
Yes	56		64
No	31		36

Cirrhosis			
Yes	49		56
No	38		44

Child–Pugh class			
A	52		60
B	35		40

AFP level			
≤400 ng/mL	50		57
>400 ng/mL	37		43

PVTT			
Absent	60		69
Present	27		31

Tumour location			
Left	24		28
Right	41		47
Both	22		25

Tumour burden			
≤50%	52		60
>50%	35		40

Dosage of apatinib (mg)			
250	9		10
500	78		90

ECOG, Eastern Cooperative Oncology Group; AFP, alpha-fetoprotein; data are numbers of events.

**Table 3:** Adverse reactions.

Grade, *n* (%)					
Adverse events	Grades 1 (%)	Grades 2 (%)	Grades 3 (%)	Grades 4 (%)	Grades 5 (%)
Total					
Abdominal pain	41 (47)	17 (20)	2 (2)	0	0
Fever	37 (43)	9 (10)	0	0	0
Vomiting	15 (17)	2 (2)	0	0	0
Hand-foot skin reactions	9 (10)	2 (2)	4 (2)	0	0
Appetite decrease	14 (16)	0	0	0	0
Increased ALT/AST	12 (14)	3 (3)	0	0	0
Diarrhea	7 (8)	1 (1)	1 (1)	0	0
Hypertension	9 (10)	5 (6)	1	0	0
Fatigue	10 (11)	2 (2)	0	0	0
Proteinuria	12 (14)	4 (5)	3 (3)	0	0
Headache	7 (8)	1 (1)	0	0	0
Nausea	6 (7)	1 (1)	0	0	0
Oral ulcer	3 (3)	0	0	0	0
Gastrointestinal hemorrhage	2 (2)	2 (2)	0	0	0

National Cancer Institute CTCAE version 4.03 uses grades 1 through 5 to refer to the severity of the adverse events, based on general guidelines. Grade 1, mild, asymptomatic or mild symptoms, clinical or diagnostic observations only, intervention not indicated. Grade 2, moderate, minimal, local, or noninvasive intervention indicated. Grade 3, severe, medically significant but not immediately life-threatening, hospitalization or prolongation of hospitalization indicated, disabling. Grade 4, life-threatening, urgent intervention indicated. Grade 5, death, related to adverse event. CTCAE, Common Terminology Criteria for Adverse Event.

**Table 4:** Factors affecting PFS and OS.

Parameters	PFS			*P*	OS			*P*
	HR	95% CI			HR	95% CI		
		Lower	Higher			Lower	Higher	
Univariate Cox regression								
Age (>60 vs. ≤60)	0.927	0.591	1.454	0.741	1.481	0.936	2.346	0.094
Child–Pugh (A vs. B)	1.059	0.676	1.658	0.803	1.198	0.752	1.909	0.447
Cirrhosis (yes vs. no)	1.210	0.768	1.907	0.410	1.061	0.671	1.679	0.800
AFP level (≤400 ng/mL vs. >400 ng/mL)	1.155	0.737	1.808	0.530	1.954	1.204	3.171	0.007
HBV infection (yes vs. no)	2.088	1.284	3.394	0.003	1.209	0.763	1.917	0.419
Tumour burden (≤50% vs. >50%)	13.089	3.898	13.089	<0.001	6.576	3.756	11.512	<0.001
PVTT (absent vs. present)	6.081	3.378	10.946	<0.001	4.854	2.787	8.455	<0.001

Multivariate Cox regression								
Age (>60 vs. ≤60)	0.726	0.441	1.195	0.209	2.206	1.306	3.728	0.003
Child–Pugh (A vs. B)	1.012	0.594	1.724	0.964	1.577	0.943	2.638	0.082
Cirrhosis (yes vs. no)	1.376	0.831	2.278	0.215	1.360	0.798	2.317	0.258
AFP level (≤400 ng/mL vs. >400 ng/mL)	1.233	0.711	2.139	0.456	0.923	0.544	1.564	0.765
HBV infection (yes vs. no)	1.370	0.787	2.386	0.265	1.313	0.750	2.300	0.341
Tumour burden (≤50% vs. >50%)	19.155	9.255	39.647	<0.001	15.484	7.865	30.484	<0.001
PVTT (absent vs. present)	15.714	7.208	34.257	<0.001	12.731	6.188	26.194	<0.001

OS, overall survival; HR, hazard ratio; CI, confidence interval; PFS, progression-free survival; AFP, alpha-fetoprotein; HBV, hepatitis B virus; PVTT, portal vein tumor thrombus.

## Data Availability

The clinical data were obtained from the Interventional Department of the First Affiliated Hospital of Zhengzhou University. The data used to support the findings of this study are available from the corresponding author upon request.

## Abbreviations

TACE: Transcatheter arterial chemoembolization
ATO: Arsenic trioxide
aTACE: Arsenic trioxide transcatheter arterial chemoembolization
CTCAE: Common Terminology Criteria for Adverse Event
PVTT: Portal vein tumor thrombus
VEGFR-2: Vascular endothelial growth factor receptor 2
CT: Computed tomography
HCC: Hepatocellular carcinoma
PFS: Progression-free survival
OS: Overall survival
AFP: Alpha-fetoprotein
ALB: Albumin
ALT: Alanine aminotransferase
AST: Aspartate aminotransferase
TBIL: Total bilirubin
mRECIST: Modified Response Evaluation Criteria in Solid Tumors
VEGF: Vascular endothelial growth factor.
